# Protein Intake, Nutritional Status and Outcomes in ICU Survivors: A Single Center Cohort Study

**DOI:** 10.3390/jcm8010043

**Published:** 2019-01-04

**Authors:** Peter J.M. Weijs, Kris M. Mogensen, James D. Rawn, Kenneth B. Christopher

**Affiliations:** 1 Department of Nutrition and Dietetics, Amsterdam University Medical Centers, VU University, 1081 HV Amsterdam, The Netherlands; p.weijs@vumc.nl; 2 Faculty of Sports and Nutrition, Amsterdam University of Applied Sciences, 1067 SM Amsterdam, The Netherlands; 3 Department of Nutrition, Brigham and Women’s Hospital, Boston, MA 02115, USA; kmogensen@bwh.harvard.edu; 4 Department of Surgery, Brigham and Women’s Hospital, Boston, MA 02115, USA; jrawn@bwh.harvard.edu; 5 The Nathan E. Hellman Memorial Laboratory, Division of Renal Medicine, Brigham and Women’s Hospital, Boston, MA 02115, USA

**Keywords:** protein, malnutrition, critical care, mortality, outcomes, hospital readmission, ICU Survivors

## Abstract

Background: We hypothesized that protein delivery during hospitalization in patients who survived critical care would be associated with outcomes following hospital discharge. Methods: We studied 801 patients, age ≥ 18 years, who received critical care between 2004 and 2012 and survived hospitalization. All patients underwent a registered dietitian formal assessment within 48 h of ICU admission. The exposure of interest, grams of protein per kilogram body weight delivered per day, was determined from all oral, enteral and parenteral sources for up to 28 days. Adjusted odds ratios for all cause 90-day post-discharge mortality were estimated by mixed- effects logistic regression models. Results: The 90-day post-discharge mortality was 13.9%. The mean nutrition delivery days recorded was 15. In a mixed-effect logistic regression model adjusted for age, gender, race, Deyo-Charlson comorbidity index, acute organ failures, sepsis and percent energy needs met, the 90-day post-discharge mortality rate was 17% (95% CI: 6–26) lower for each 1 g/kg increase in daily protein delivery (OR = 0.83 (95% CI 0.74–0.94; *p* = 0.002)). Conclusions: Adult medical ICU patients with improvements in daily protein intake during hospitalization who survive hospitalization have decreased odds of mortality in the 3 months following hospital discharge.

## 1. Introduction

A hallmark of critical illness is muscle wasting related to a dramatic increase in muscle protein catabolism in the setting of inflammation [1]. Catabolic critical illness results in a protracted and dramatic loss of nitrogen and reduces exogenous amino acid deposition into endogenous proteins [2]. Nutritionally compromised hospitalized patients commonly present with sarcopenia [3]. Inflammation related endogenous skeletal muscle protein catabolism that can quickly progress to severe muscle atrophy [4]. In the critically ill, muscle mass loss and decreased strength are common complications [5].

Protein intake recommended for healthy adults is 0.8 g/kg per day. While the most common recommended protein intake in the critically ill is 1.5 g/kg per day [6,7], up to 1.5–2.5 g protein/kg per day may be most advantageous [7,8]. ICU patients commonly receive less than even the lowest guideline recommendation regarding adequate protein intake [7,9,10,11]. Recent trials [12,13,14,15] demonstrate that “calorie-supplemented, protein-deficient nutrition” [4] does not improve clinical outcomes. Early energy provision of 70–80% of measured energy expenditure appears to be associated with improved outcome [16,17]. Although the correct amount of protein to provide critically ill patients is unknown, higher than 1.2 g/kg is associated with reduced mortality in non-septic non-energy overfed ICU patients [16]. A recent large observational study suggests achieving ≥80% of prescribed protein intake in the ICU is associated with decreased mortality [18].

Although short term survival is studied in the critically ill regarding protein delivery [18], post-hospital discharge outcomes in such ICU survivors relative to protein intake and nutrition status is not known. In patients who survive critical illness, pre-existing malnutrition may be a risk factor for adverse events following hospital discharge [19]. As ICU patients with low protein delivery [18] and with malnutrition [20] have elevated in-hospital mortality, we studied the association between increases in daily protein delivery in malnourished critically ill patients would be associated with lower 90-day mortality following hospital discharge. We hypothesized that in hospital survivors who received critical care, those with higher daily protein delivery would have a lower risk of post discharge mortality and this effect would be magnified in patients with pre-existing malnutrition.

## 2. Materials and Methods

### 2.1. Data Sources

We obtained administrative and laboratory data from critically ill medical patients admitted to the Brigham and Women’s Hospital (BWH), an academic medical center in Boston with 777 beds. Patient data was collected by the Research Patient Data Registry (RPDR) a clinical data warehouse for patient records at BWH that has been utilized and validated in other studies [20]. The Partners Human Research Committee Institutional Review Board granted approval for the study. Between 2004 and 2012 there were 801 unique patients ≥18 years, assigned the CPT code 99291 (critical care, first 30–74 min) [21] who had nutrition risk assessed, protein intake measured, and survived to hospital discharge.

### 2.2. Exposure of Interest and Comorbidities

All medical ICU patients at BWH are screened by a registered dietitian (RD). Patients at risk for malnutrition are then evaluated by an RD with a structured objective assessment. The exposure of interest, grams of protein per kilogram body weight delivered per day, was determined by an RD from all oral, enteral and parenteral sources for up to 28 days. We included all nutrition data collected by an RD in the ten days prior to ICU admission to two days following an ICU discharge. The determination of nutrition status is described in detail previously [20,22]. The RD determines the diagnosis of malnutrition based on literature [23,24], clinical judgment, and on data related to inadequate nutrient intake, muscle wasting, subcutaneous fat wasting and unintended weight loss [22]. As in our prior study, we categorized nutrition diagnoses a priori into four groups of increasing severity: malnutrition absent, at risk for malnutrition, non-specific malnutrition, or any protein-energy malnutrition [22]. Patients that meet criteria for non-specific malnutrition have risk factors for malnutrition (insufficient intake of energy, protein, and micronutrients) in addition to metabolic stress and/or obvious signs of malnutrition (muscle and/or subcutaneous fat wasting) without supporting biochemical or anthropometric data. Patients that meet criteria for protein-energy malnutrition have a combination of disease-related weight loss, obvious muscle wasting, peripheral edema, inadequate energy or protein intake and are considered underweight by percent ideal body weight [25]. Patients with absence of malnutrition are diagnosed by an RD as well-nourished and not at risk for malnutrition.Race was self-determined or determined by the patient’s family or healthcare proxy. The Deyo-Charlson comorbidity index was determined using validated ICD-9 coding algorithms [26]. To define sepsis we used the presence of the ICD-9 codes 038, 785.52, 995.91, or 995.92 in the three days before ICU admission to the 7 days after ICU admission [27]. The vasopressors/inotropes covariate is the prescription of any vasopressor or inotrope in the three days prior to ICU admission to the seven days after ICU admission [20].

The number of acute organ failures was a combination of ICD-9-CM and CPT codes relating to acute organ dysfunction assigned from three days prior to ICU admission day to the 30 days after [28,29]. Intubation was defined as the presence of ICD-9 codes mechanical ventilation (96.7×) following hospital admission [30]. Acute kidney injury is the RIFLE class Injury or Failure taking place in the three days prior to ICU admission and the seven days after ICU admission [31]. Chronic kidney disease is <60 mL/min glomerular filtration rate calculated from the Modification of Diet in Renal Disease (MDRD) equation using pre-hospital or hospital admission creatinine as baseline [32]. Changes from the expected hospital length of stay (LOS) are determined by subtracting the actual LOS from the geometric mean LOS for each Diagnostic Related Grouping (DRG) a classification system for hospital cases [33]. The geometric mean LOS is the United States national mean LOS for each DRG per the Centers for Medicare & Medicaid Services [34].

### 2.3. End Points

Our primary outcome was all cause 90-day post-discharge mortality. Mortality status for the study cohort was obtained from the United States Social Security Administration Death Master File. Mortality determination via the Death Master File is validated in our administrative database [21]. The entire cohort had mortality status present for the year following hospital discharge. The censoring date was 15 March 2012. All patients in the cohort had at least 90-day follow-up after hospital discharge or expired before the 90-days post-hospitalization.

### 2.4. Power Calculations and Statistical Analysis

90-day post-discharge mortality rate was 9.6% in our prior study nutrition in critical illness [35]. From these data, we assumed that 90-day post-discharge mortality would be 6.0% greater in patients with <1.0 g/kg/day protein delivery compared to those with >1.0 g/kg/day protein delivery. With a power of 80% and an alpha error level of 5%, the sample size required for the 90-day post-discharge mortality outcome was 393 patients with >1.0 g/kg/day protein delivery and 393 patients with <1.0 g/kg/day protein delivery.

We described categorical covariates via frequency distribution, and compared variables in outcome groups with chi-square testing. We compared continuous variables across outcome groups using one-way analysis of variance (ANOVA) or the Kruskal–Wallis test. We utilized mixed-effect logistic regression models [36,37] which contain both fixed and random-effects for analysis of the association between g/kg/day protein delivery and 90-day post-discharge mortality by use of the command xtmelogit [38] in STATA 14.1 MP (College Station, TX, USA). The dates of protein delivery of individual patients were used as the random effect. We assessed the following covariates for confounding: age, gender, race, Deyo-Charlson comorbidity index, energy delivery, nutrition status, acute organ failure, sepsis, metastatic malignancy, acute kidney injury and chronic kidney disease. We selected the final model confounders by analyzing the maximum model and then conducting backward elimination of covariates with a *p* > 0.10. The final model had an independent covariance structure of the random effects; a fixed effect for age, gender, race, Deyo-Charlson comorbidity index, energy delivery, nutrition status, acute organ failure and sepsis; and gaussian-distributed random intercepts and slopes. We then used mixed-effect linear regression to analyze the association between the change in daily protein delivery and hospital LOS where random effects accounted for the dates of protein delivery within individual patients. Data visualization was performed utilizing the DistillerSR Forest Plot Generator (Evidence Partners, Ottawa, ON, Canada). *p*-values presented are two-tailed with *p* < 0.05 considered to be significant. We utilized STATA 14.1MP (College Station, TX, USA) for all analyses.

## 3. Results

**Figure 1:**
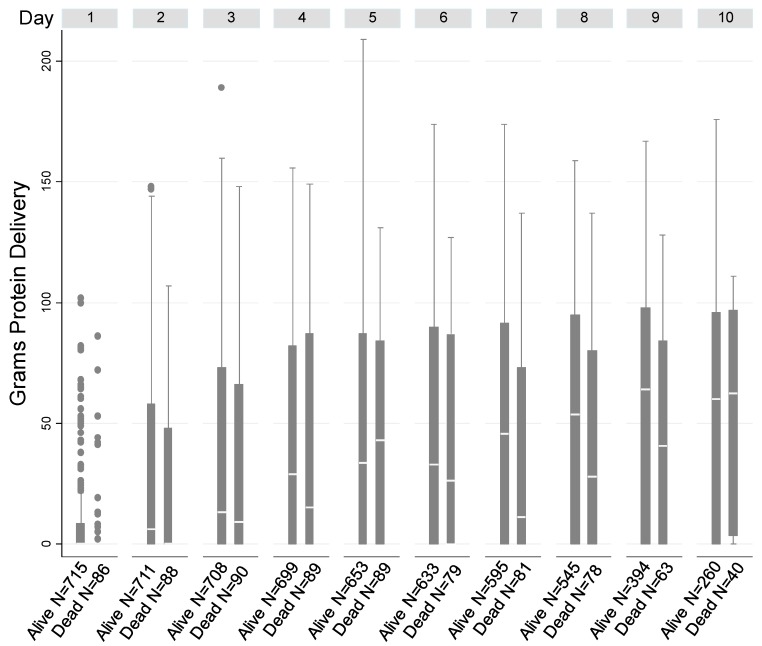
Box plots of daily protein delivery up to 10 days in patients who did and did not survive to 90-day post-discharge mortality, showing the median (white line), the third quartile (Q3) and first quartile (Q1) range of the data and data outliers (observations outside the 9–91 percentile range).

Patient characteristics were stratified according to 90-day post-discharge mortality (Table 1). In our cohort the mean (SD) age was 62.3 (16.6) years, 55.3% were male and 78.9% were white. The majority of patients were assessed by an RD within 24 hours of ICU admission (median [IQR] time between ICU admission and RD assessment was 0 [1, 1] days). The mean number of recorded nutrition delivery days was 15. A total of 8735 days with protein intake were determined. The mean peak protein daily delivery was 0.32 g protein/kg. Figure 1 shows protein delivery over time in the cohort.

The mean (SD) length of hospital stay was 22.1 (15.2) days. The 30-, 90- and 365-day post-discharge mortality rates were 7.1%, 13.9%, and 24.5%, respectively. Age, race, Deyo-Charlson comorbidity index, malnutrition, the acute organ failure score and chronic kidney disease are significantly associated with 90-day post-discharge mortality (Table 1).

### Primary Outcome

Higher protein delivery in ICU survivors was associated with lower 90-day post-discharge mortality. In our mixed-effect logistic regression model adjusted for age, gender, race, Deyo-Charlson comorbidity index, energy delivery, nutrition status, acute organ failure, sepsis and the random-effects structure, the 90-day post-discharge mortality rate decreased by 18% for each 1 g/kg/day elevation in protein delivery following ICU admission (OR 0.82, 95% CI 0.73–0.92). Following stratification for nutrition status, the same multivariable mixed-effect logistic regression model shows the 90-day post-discharge mortality rate significantly decreased for each 1 g/kg elevation in daily protein delivery. Specifically, in patients diagnosed with malnutrition (*n* = 473), 90-day post-discharge mortality rate was 30% (95% CI: 6–26) less for each 1 g/kg elevation in daily protein delivery following ICU admission (OR = 0.70 (95% CI 0.61–0.81; *p* < 0.001)).

**Figure 2:**
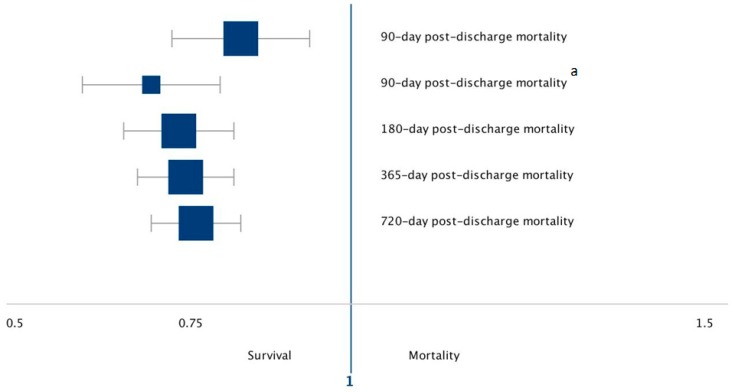
Forest plot of post-discharge mortality associated for each 1 g/kg/day increase in protein delivery in our study cohort. Odds ratios with corresponding 95% CIs shown of the individual outcomes under study. ^a^ Restricted to patients diagnosed with Malnutrition.

We next determined if protein delivery was associated with longer term mortality outcomes. When patients were evaluated using a multivariable mixed-effect logistic regression model with the same covariates and structure as the primary outcome model, 180-day post-discharge mortality was significantly reduced for each 1 g/kg elevation in daily protein delivery (OR 0.74, 95% CI 0.67–0.83; *p* < 0.001). When patients were evaluated using the multivariable mixed-effect logistic regression model with the same covariates and structure as the primary outcome model, the 365- and 720-day post-discharge mortality rate was significantly reduced for each 1 g/kg elevation in daily protein delivery (OR 0.76, 95% CI 0.69–0.83; *p* < 0.001), (OR 0.77, 95% CI 0.71–0.84; *p* < 0.001) respectively (Table 2, Figure 2). All estimates are for each 1 g/kg/day increase in protein delivery.

Finally we examined the association between protein delivery and hospital length of stay. In a mixed-effect linear regression model adjusted for age, gender, race, Deyo-Charlson comorbidity index, energy delivery, nutrition status, acute organ failure, sepsis and the random-effects structure, the actual hospital length of stay (LOS) was reduced by 0.87 days (95% CI −1.7 to −0.10) compared with the average LOS for the DRG for each 1 g/kg/day increase in protein delivery (*p* = 0.028).

## 4. Discussion

In our study, we examined the association between protein delivery in critical illness survivors and post-hospital discharge outcomes. We demonstrate that improvement in daily protein delivery is associated with a significant decrease in the odds of post-discharge hospital mortality. We also show that patients with improved daily protein delivery have decreased hospital length of stay.

Long-term morbidity and mortality are substantial problems in ICU survivors [39]. Adverse events following discharge from an ICU admission are known to include comorbidity, illness severity and organ failures [40]. In critical illness survivors, the risk factors for outside hospital adverse events are not well known. Our results show an association between higher daily protein delivery during hospitalization and improved post-discharge mortality in critical illness survivors. While our observational study cannot be interpreted as causal, the protein delivery-post-discharge outcome association has biologic plausibility.

Our study may have limitations due to its observational design. Causality is limited and as such our observations should be considered hypothesis generating. As protein intake in our cohort is only collected in patients considered to be at least at risk for malnutrition, ascertainment bias may be present. Unmeasured variables may influence outcomes following hospitalization independent of protein delivery, which can result in bias. We are unable to adjust for gut failure which associated with lower nutrition tolerance, lower protein intake and contributes to adverse outcomes [41]. Though ICD-9 code assignment data is collected from individual provider encounters, determination of covariates via ICD-9 codes may not completely capture the true incidence or prevalence. Finally, residual confounding may remain despite multivariable adjustment which may contribute to observed estimates.

Our study has several strengths. We utilize mixed-effects models to incorporate multiple measures and sources of protein intake over a total of 8735 days which allow for more complete information to be captured in parameter estimations. Mixed-effects models are ideal for such longitudinal data because each patient may have an unequal number of repeated measures. Additionally, a highly trained RD utilized weight loss history, clinical assessment, anthropometric metrics and protein intake to make an in person assessment of nutritional risk and daily protein delivery. Finally, the Master Death File is shown to capture post discharge mortality in our cohort accurately [21].

## 5. Conclusions

Our data show that in critically ill patients who survive hospitalization, higher daily protein delivery during hospitalization is associated with decreased mortality following hospital discharge. Increasing protein delivery following the acute phase of illness may be a strategy to assist with critical illness recovery and longer term outcomes.

## Figures and Tables

**Table 1:** Clinical and demographic characteristics of the study cohort (*n* = 801).

Characteristics	Alive at 90-Days Post-Discharge	Expired by 90-Days Post-Discharge	Total	*p*-Value	Unadjusted OR (95%CI) for 90-Day Post-Discharge Mortality
	690	111	801		
*Age-Mean ± SD*	61.1 ± 16.7	69.8 ± 14	62.3 ± 16.6	<0.001 *	1.04 (1.02, 1.05)
*Male Gender-No. (%)*	384 (56)	58 (52)	442 (55)	0.50	0.87 (0.58, 1.30)
*Non-White Race-No. (%)*	154 (22)	15 (14)	169 (21)	0.035	0.54 (0.31, 0.96)
*Deyo-Charlson Index-No. (%)*				0.003	
0	182 (26.38)	15 (13.39)	197 (24.56)		1.00 (Referent)
1–2	440 (63.77)	75 (66.96)	515 (64.21)		2.07 (1.16, 3.70)
≥3	68 (9.86)	22 (19.64)	90 (11.22)		3.93 (1.92, 8.01)
*Acute Organ Failures-No. (%)*				0.030	
0	86 (12)	16 (14)	102 (13)		1.00 (Referent)
1	217 (31)	29 (26)	246 (31)		0.72 (0.37, 1.39)
2	192 (28)	26 (23)	218 (27)		0.73 (0.37, 1.43)
3	136 (20)	20 (18)	156 (19)		0.79 (0.39, 1.61)
≥4	59 (9)	20 (18)	79 (10)		1.82 (0.87, 3.80)
*Sepsis-No. (%)*	138 (20)	28 (25)	166 (21)	0.21	1.35 (0.85, 2.15)
*Intubation-No. (%)*	400 (58)	60 (54)	460 (57)	0.44	0.85 (0.57, 1.28)
*Acute Organ Failure Score-Mean ± SD*	10.2 ± 4.4	11.4 ± 4.6	10.4 ± 4.5	0.0082 *	1.06 (1.02, 1.11)
*Vasopressors/Inotropes-No. (%)*	264 (38)	51 (46)	315 (39)	0.12	1.37 (0.92, 2.05)
*Metastatic Malignancy-No. (%)*	296 (43)	58 (52)	354 (44)	0.066	1.46 (0.97, 2.18)
*Acute Kidney Injury-No. (%)* **^†^**	55 (10)	6 (7)	61 (10)	0.381	0.68 (0.28, 1.63)
*Chronic Kidney Disease-No. (%)* **^††^**	169 (31)	42 (49)	211 (33)	0.001	1.23 (1.06, 1.41)
*Malnutrition-No. (%)*				0.035	
At Risk for Malnutrition	291 (43)	33 (30)	324 (41)		1.00 (Referent)
Non-Specific Malnutrition	343 (51)	65 (60)	408 (52)		1.67 (1.07, 2.61)
Protein-Energy Malnutrition	45 (7)	11 (10)	56 (7)		2.16 (1.02, 4.57)

Data presented as *n* (%) unless otherwise indicated. *p* values determined by chi-square unless designated by (*) then *p* value determined by ANOVA. **^†^** Data available to determine Acute Kidney Injury in 638 patients. **^††^** Data available to determine Chronic Kidney Disease was present in 638 patients.

**Table 2:** Associations between Protein Delivery and post-hospital mortality outcomes.

Outcome	OR	95% CI	*p*-Value	Association for Each 1 g/kg/Day Increase in Protein Delivery
90-day post-discharge Mortality				
*Full Cohort (n = 801)*	0.83	0.74–0.94	0.002	17% decrease odds of death
*Malnutrition (n = 473)*	0.70	0.61–0.81	<0.001	30% decrease odds of death
180-day post-discharge Mortality				
*Full Cohort (n = 801)*	0.74	0.67–0.83	<0.001	26% decrease odds of death
365-day post-discharge Mortality				
*Full Cohort (n = 801)*	0.76	0.69–0.83	<0.001	24% decrease odds of death
720-day post-discharge Mortality				
*Full Cohort (n = 801)*	0.77	0.71–0.84	<0.001	23% decrease odds of death

All estimates were produced via a mixed-effect logistic regression model adjusted for age, gender, race, Deyo-Charlson comorbidity index, energy delivery, nutrition status, acute organ failure, sepsis and the random-effects structure.
